# Effectiveness and safety of Guilu Erxian Glue (a traditional Chinese medicinal product) for the treatment of postmenopausal osteoporosis: A protocol for systematic review and meta-analysis

**DOI:** 10.1097/MD.0000000000020773

**Published:** 2020-07-17

**Authors:** Yuhao Si, Yao Yao, Yong Ma, Yang Guo, Heng Yin

**Affiliations:** a The First School of Clinical Medicine; b School of Nursing; c College of Basic Medicine, Nanjing University of Chinese Medicine, Nanjing; d Department of Traumatology & Orthopedics, Wuxi Affiliated Hospital of Nanjing University of Chinese Medicine, Wuxi, China.

**Keywords:** Guilu Erxian Glue, postmenopausal osteoporosis, postmenopausal women, protocol, systematic review

## Abstract

**Background:**

The prevalence of postmenopausal osteoporosis is rapidly rising, with a high proportion of patients who are vulnerable to fractures and other chronic symptoms. The commonly applied Western medicine lacks cost-efficiency and tends to have various potential side effects. Guilu Erxian Glue (GEG), an orally taken traditional Chinese medicinal product, has been advocated to manage osteoporosis for a long period. However, the evidence of the efficacy of GEG on osteoporosis seems to be less convincing and conclusive. Therefore, the purpose of this study is to assess the effectiveness and safety of GEG on postmenopausal osteoporosis.

**Methods:**

We will implement a systematic review and meta-analysis following the requests of the preferred reporting items for systematic reviews and meta-analyses protocols (PRISMA-P). The PubMed database, Scopus, Embase, Web of Science, Cochrane Library, China National Knowledge Infrastructure, Wanfang Database, VIP database, Sinomed, and grey literature sources will be retrieved for eligible studies. Randomized controlled trials investigating GEG in women with postmenopausal osteoporosis employing osteoporosis-associated outcomes measures will be eligible for inclusion. Two reviewers will independently complete the study selection procedure, data extraction, and risk of bias assessment. A third reviewer will mediate when irreconcilable discrepancies occur. Should sufficient clinical homogeneity exist, a meta-analysis will be conducted. Additionally, we will utilize the Grading of Recommendations Assessment, Development and Evaluation to assess the strength of quantitative outcomes.

**Results:**

The current systematic review and meta-analysis will provide the effectiveness and safety of GEG on postmenopausal osteoporosis.

**Conclusion:**

The study will offer high-quality and explicit evidence for applying GEG on the treatment of postmenopausal osteoporosis.

**OSF Registration:**

DOI 10.17605/OSF.IO/JCVBH.

## Abstract 2

Supplemental Digital Content is available in the text

## 1 Introduction

A significant number of postmenopausal women worldwide suffer from metabolic bone disease osteoporosis, and it has become a public health issue as a result of a higher risk of fractures and subsequent complications.^[1]^ It is suggested that the occurrence of fractures in older adults induces a rise in mortality, morbidity, and social-economic burdens.^[2,3]^ In 2005, approximately 1.45 million women over the age of 50 experienced fractures in America.^[4]^ Furthermore, a survey conducted in 2017 evaluated osteoporosis knowledge of 1012 postmenopausal American women over 50, and involved those who were diagnosed with osteoporosis and those who were not. Among those participants, 501 women stated that they had been diagnosed with osteoporosis.^[5]^ Notably, the risks of osteoporosis and related fractures have increasingly risen in recent years due to the aging population and extended life expectancy in numerous developed countries.

No gold standard treatment for postmenopausal osteoporosis is currently released. Pharmacological interventions, such as oral or intravenous bisphosphonates, denosumab, menopause hormone therapy, and raloxifene, are recommended for postmenopausal women.^[6]^ Nevertheless, Western medicine therapy is an expensive choice with uncertain clinical effects and even leads to some potential adverse reactions such as fever, arthralgia myalgia, hypocalcemia, skin infections, etc.^[7–10]^ Consequently, it is of extraordinary importance to detect alternative treatments with higher cost-efficiency and fewer side effects for osteoporosis.

Guilu Erxian Glue (GEG) is a typical traditional Chinese medicinal product with multiple components, primarily originating from processed tortoise shells and antlers.^[11]^ GEG has been widely applied in China for the treatment and prevention of osteoporosis for hundreds of years with minimum side effects (xerostomia).^[12]^ While some randomized controlled trials (RCTs) have investigated GEG's anti-osteoporosis impacts and safety, some RCTs tend to possess insufficient sample sizes and methodological qualities.^[13–15]^ Furthermore, no systematic review and meta-analysis for the effectiveness and safety of GEG on osteoporosis has been implemented thus far. Therefore, the evidence for employing GEG in the treatment of postmenopausal osteoporosis is less convincing and conclusive. The purpose of this prospective study is to investigate the effectiveness and safety of GEG in the management of postmenopausal osteoporosis through compiling and analyzing the existing research data in a systematic review and meta-analysis approach.

## 2 Methods

### 2.1 Protocol and registration

This study was prospectively registered in the Open Science Framework (OSF) with a DOI: 10.17605/OSF.IO/JCVBH. We direct this systematic review protocol in accordance with the preferred reporting items for systematic review and meta-analysis protocols (PRISMA-P) checklist.^[16]^

### 2.2 Inclusion criteria

Studies that meet the following criteria will be enrolled in our review:

1. The study design was an RCT.
2. The published language was in English or Chinese.
3. The target participants were postmenopausal women diagnosed with osteoporosis, utilizing recognized diagnostic criteria.
4. One of the interventions was orally taken GEG, regardless of the dose, frequency, and duration.
5. The trial compared GEG with other interventions, such as Western medicine, aerobic exercise, education, calcium supplements, and lifestyle improvements.
6. Osteoporosis-related outcomes were reported, such as bone mineral density, bone formation or resorption biomarkers, falls and fractures. Secondary outcomes might also be delivered, such as quality of life and adverse events.

### 2.3 Exclusion criteria

Studies that meet the following criteria will be excluded from our review:

1. Participants diagnosed with idiopathic osteoporosis.
2. A duplicate report.
3. Necessary data was unavailable to be received from corresponding authors.

### 2.4 Search strategy

The following electronic databases will be retrieved for potential articles, comprising of the PubMed database, Scopus, Embase, Web of Science, Cochrane Library and Chinese databases, namely, China National Knowledge Infrastructure, Wanfang Database, the VIP database, and Sinomed. Each database will be searched from their inception up to May 2020. We will also conduct a manual search in the library of the Nanjing University of Chinese Medicine and Duquesne University in case of any missing literature. Additionally, we will hand-search all the related references in potential articles for further identification. The MeSH terms applied for retrieval are closely associated with “Guilu Erxian Glue,” “postmenopausal osteoporosis,” and “randomized controlled trial,” and the detailed electronic search strategy is accessible in online supplemental files (Appendix 1).

### 2.5 Selection process

**Figure 1:**
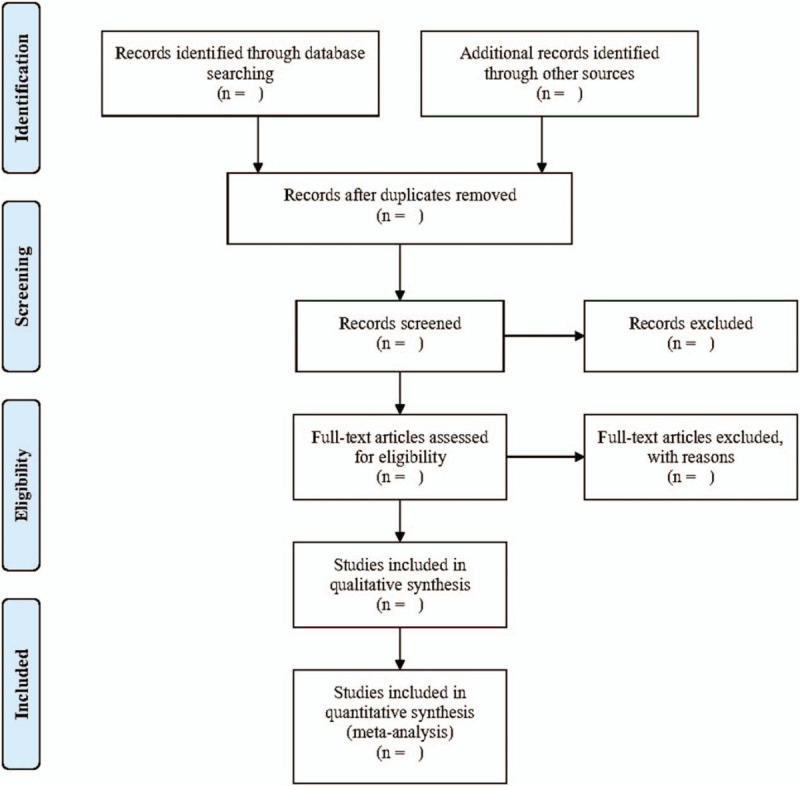
Study selection flow diagram.

Two authors (YS and YY) will independently screen the titles and abstracts of potential articles yielded in the search process against the eligibility criteria. They will then read the entire contents of articles and their relevant references for final collection. Any conflicts of trial identifications will be resolved through open discussions or judged by an arbitrator (YM). Moreover, relevant information (including the reasons for study exclusion) generated in this procedure will be recorded in a Microsoft Excel spreadsheet (Microsoft Corporation, Redmond, WA). The preferred reporting items for systematic reviews and meta-analyses (PRISMA) flowchart (Fig. 1) will be filled out after the completion of screening studies to offer specific information.

### 2.6 Data extraction and management

Two authors (YY and YG) will independently complete the data extraction and collection of the studies that have passed the study selection procedure. A brief and structured electronic form embodying the following information will be completed.

1. Study characteristics: author name, publication year, sample size, trial location, contact information.
2. Participant characteristics: age, sex, geographic population, number in each group, health status (with/without osteoporosis), diagnosis criteria if provided.
3. Study design: how the authors implemented randomization, allocation concealment, blinding, and the way in which they dealt with the loss to follow-up.
4. Interventions: dose, frequency and duration of GEG intervention, and other interventions, routes of drug administration.
5. Outcomes: osteoporosis-associated symptoms, fractures and falls, bone mineral density, bone formation and resorption biomarkers, quality of life, adverse reactions.

Any discrepancies that cannot be resolved through discussion will be decided by a third reviewer (HY). Furthermore, all the recorded information will be double-checked by a separate author (YM).

### 2.7 Assessment of risk of bias in individual study

Two reviewers (YS, YY) will independently evaluate the internal validity of eligible studies applying the Cochrane Risk of Bias tool.^[17]^ Any discrepancies will be mediated by a third author (HY). Each domain will be rated by high, low, or unclear risk of bias in line with the extracted data. We will also assess the between-reviews agreement utilizing Cohen *k*.^[17]^

### 2.8 Assessment of heterogeneity

Clinical heterogeneity is the first step in evaluation, which is done by comparing each study's characteristics based on professional knowledge. We will explore intervention heterogeneity among included trials concerning the classifications of dosage, routes of administration, duration, and drug/herbal providers. Statistical heterogeneity will not be appraised and a narrative review will be delivered if notable clinical heterogeneity exists. Should clinical homogeneity exist but it is not significant, *I*^2^ will be utilized to evaluate the statistical heterogeneity and interpreted according to the Cochrane Handbook for Systematic Review of Interventions (0– 40%, 0–60%, 50–90%, and 75–100% represents low, moderate, substantial, and considerable heterogeneity).^[17]^ Meanwhile, the *P* value from the chi-squared test will be examined, employing a *P* value <.10 to ascertain notable heterogeneity.

### 2.9 Data synthesis

The process of retrieving and gathering data will be recorded on a PRISMA flow chart. Meta-analyses will be administered to quantitatively incorporate data when studies possess clinical homogeneity and low-level statistical heterogeneity.^[17]^ Groups of trials in which heterogeneity is rated low (*I*^2^ < 50%) will be pooled through using a fixed effects model. Otherwise, a random effects model will be chosen. Continuous data will be analyzed by applying standardized mean difference or mean difference (with 95% CIs) if several measurements are involved. Dichotomous data will be merged through utilizing risk ratios with 95% CIs. The results will be exhibited using forest plots and well-structured tables. Additionally, incorporated intervention effects will be estimated by employing a weighted mean of the intervention effects measured in the single studies.

### 2.10 Sensitivity analysis

We will examine the following factors to investigate whether modifying some conditions would influence the meta-analysis outcomes: recruiting data only from studies that are published in English; omitting data from studies that arerated as having a high risk of bias.

### 2.11 Publication bias

We will search for grey literature on the online clinical trial registry databases and contact authors of relevant registries to obtain unpublished data. Furthermore, we will employ the funnel plot and statistical tests (such as the Egger test and the Begg test) to assess the publication bias.^[18]^

### 2.12 Assessment of quantitative outcomes

The quality of the evidence will be judged through using Grading of Recommendations, Assessment, Development and Evaluation.^[17,19]^ The quality will be categorized as high, moderate, low, or very low.

### 2.13 Patient and public involvement

There is no necessity to state patient and public involvement since the current study is a protocol for a systematic review and meta-analysis.

### 2.14 Amendments

If any amendment regarding the current protocol is made, we will deliver the contents, timing, and rationale of the amendment in the published paper and OSF.

### 2.15 Ethics and dissemination

No ethical approval is required for this study. Our findings will be published in a peer-reviewed journal and presented at national and provincial conferences.

## 3 Conclusion

China has been into the early stage of an aging society, which may lead to an upward trend of postmenopausal osteoporosis occurrence. This situation will increase the continuing economic burden for the individual family and the entire society. The universal application of traditional medicinal products, such as GEG, is considered to be a potential approach to decrease the economic burden due to its high cost-efficiency. Since the formula and function of GEG are originated from the ancient experience of traditional Chinese medicine, more detailed and precise evidence is required prior to further clinical use. Therefore, the current study is conceived and will be conducted to provide evidence-based information on GEG for ameliorating postmenopausal osteoporosis.

## Acknowledgments

The authors would like to express their gratitude to Professor Xi Huang, the “Outstanding Youth” at the Nanjing University of Chinese Medicine, for his financial support from the project of integration of Chinese and Western medicine.

## Author contributions

All authors have critically read and approved the final version of this manuscript.

**Conceptualization**: Yuhao Si, Heng Yin.

**Formal analysis:** Yuhao Si, Yong Ma.

**Funding acquisition**: Heng Yin.

**Methodology:** Yuhao Si, Yao Yao, Yang Guo, Yong Ma, Heng Yin.

**Software:** Yuhao Si, Yao Yao.

**Supervision**: Heng Yin.

**Validation:** Heng Yin.

**Writing – original draft**: Yuhao Si, Yao Yao.

**Writing – review & editing**: Yuhao Si, Yao Yao, Yang Guo, Yong Ma, Heng Yin.

## Supplementary Material

Supplemental Digital Content
