# Supplementary material for: Effectiveness and safety of Guilu Erxian Glue (a traditional Chinese medicinal product) for the treatment of postmenopausal osteoporosis: A protocol for systematic review and meta-analysis
Source: Medicine (Baltimore). 2020 Jul 17;99(29):e20773. doi: 10.1097/MD.0000000000020773 (PMC7373594; doi:10.1097/MD.0000000000020773)
Supplement: Supplemental Digital Content [file medi-99-e20773-s001.docx]

**Appendix 1. Search strategy for PubMed**

| No. | Search terms |  |
| --- | --- | --- |
| #1 | Guilu Erxian Glue [MeSH Terms] |  |
| #2 | Guilu Erxian Jiao [MeSH Terms] |  |
| #3 | Guilu Erxian decoction [MeSH Terms] |  |
| #4 | Guilu Erxian [MeSH Terms] |  |
| #5 | Guilu Erxian Glue [Title/Abstract] |  |
| #6 | Guilu Erxian Jiao [Title/Abstract] |  |
| #7 | Guilu Erxian decoction [Title/Abstract] |  |
| #8 | Guilu Erxian [Title/Abstract] |  |
| #9 | #1 OR #2 OR #3 OR #4 OR #5 OR #6 OR #7 OR #8 |  |
| #10 | Postmenopausal osteoporosis [MeSH Terms] |  |
| #11 | Postmenopausal women [MeSH Terms] |  |
| #12 | Osteoporosis after menopause [MeSH Terms] |  |
| #13 | Osteoporosis in women [MeSH Terms] |  |
| #14 | Osteoporosis in females [MeSH Terms] |  |
| #15 | Osteoporosis in older women [MeSH Terms] |  |
| #16 | Osteoporosis in elderly women [MeSH Terms] |  |
| #17 | Osteoporosis in aged women [MeSH Terms] |  |
| #18 | Bone mass loss [MeSH Terms] |  |
| #19 | Bone mineral density [MeSH Terms] |  |
| #20 | Postmenopausal osteoporosis [Title/Abstract] |  |
| #21 | Postmenopausal women [Title/Abstract] |  |
| #22 | Osteoporosis after menopause [Title/Abstract] |  |
| #23 | Osteoporosis in women [Title/Abstract] |  |
| #24 | Osteoporosis in females [Title/Abstract] |  |
| #25 | Osteoporosis in older women [Title/Abstract] |  |
| #26 | Osteoporosis in elderly women [Title/Abstract] |  |
| #27 | Osteoporosis in aged women [Title/Abstract] |  |
| #28 | Bone mass loss [Title/Abstract] |  |
| #29 | Bone mineral density [Title/Abstract] |  |
| #30 | #10 OR #11 OR #12 OR #13 OR #14 OR #15 OR #16 OR #17 OR #18 OR #19 OR #20 OR #21 OR #22 OR #23 OR #24 OR #25 OR #26 OR #27 OR #28 OR #29 |  |
| #31 | Randomized controlled trial [MeSH Terms] |  |
| #32 | Controlled trial [MeSH Terms] |  |
| #33 | Clinical trial [MeSH Terms] |  |
| #34 | Clinical research [MeSH Terms] |  |
| #35 | Clinical report [MeSH Terms] |  |
| #36 | Clinical observation [MeSH Terms] |  |
| #37 | Randomized controlled trial [Title/Abstract] |  |
| #38 | Controlled trial [Title/Abstract] |  |
| #39 | Clinical trial [Title/Abstract] |  |
| #40 | Clinical research [Title/Abstract] |  |
| #41 | Clinical report [Title/Abstract] |  |
| #42 | Clinical observation [Title/Abstract] |  |
| #43 | #31 OR #32 OR #33 OR #34 OR #35 OR #36 OR #37 OR #38 OR #39 OR #40 OR #41 OR #42 |  |

**The search strategy will be modified as required for other electronic databases.**
